# Supplementary material for: Compensatory evolution to DNA replication stress is robust to nutrient availability
Source: Mol Syst Biol. 2025 Jun 26;21(10):1325–50. doi: 10.1038/s44320-025-00127-z (PMC12494895; doi:10.1038/s44320-025-00127-z)
Supplement: Supplementary file 14 — Expanded View Figures [file 44320_2025_127_MOESM14_ESM.pdf]

Expanded View Figures

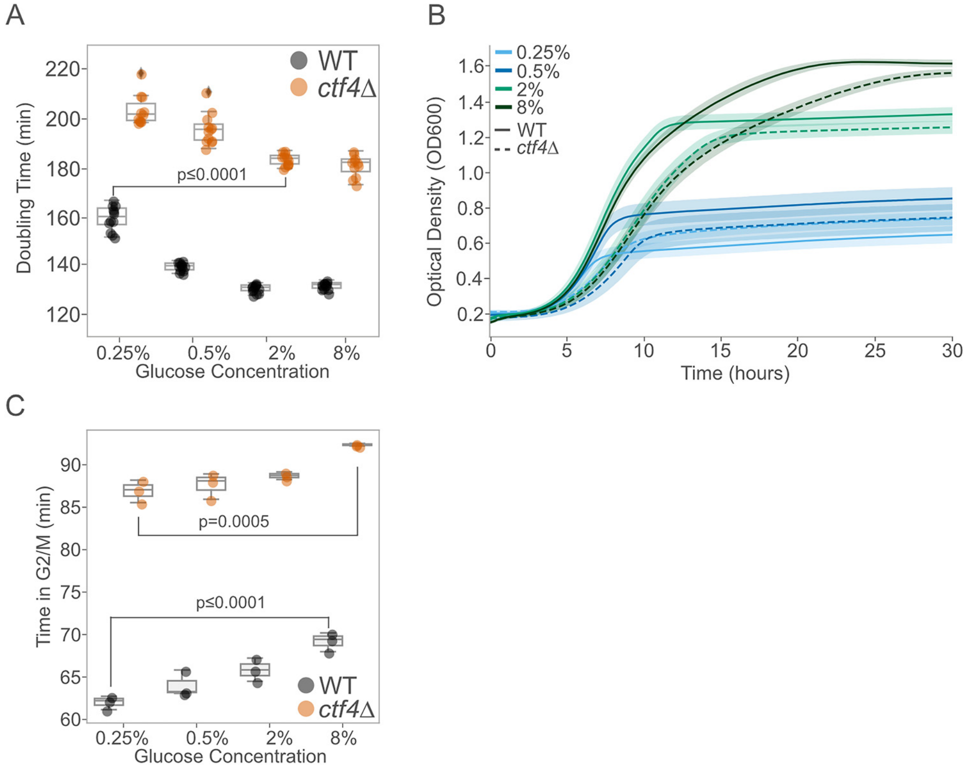

Figure EV1. Glucose concentration impacts growth dynamics in the presence of DNA replication stress.

(**A**) Population doubling time (min) of ancestral WT (black) and *ctf4Δ* mutant (orange) across different glucose concentrations ($n = 12$ biological replicates, Mann–Whitney *U* test with BH correction, *P* value $= 6.47 \times 10^{-5}$). (**B**) Growth curves of ancestral WT (solid line) and *ctf4Δ* (dashed line) over 30 h. Colors represent different glucose concentrations: light blue (0.25%), dark blue (0.5%), light green (2%), and dark green (8%). Bold lines indicate mean growth; shaded areas represent SD ($n = 12$ biological replicates). (**C**) Time spent (minutes) in G2/M phase for ancestral WT and *ctf4Δ*, across different glucose concentrations, estimated from DNA content and doubling times (see "Methods", $n = 3$ biological replicates, ANOVA Tukey's HSD) (WT 0.25% vs WT 8%, *P* value $= 2.10 \times 10^{-05}$). Box plots in (**A**, **C**) represent the median (center line), 25th and 75th percentiles (lower and upper bounds of the box), and whiskers extending to the smallest and largest values within 1.5× the IQR from the lower and upper quartiles, respectively. Detailed statistical analysis and underlying data for this figure are provided in Source Data. Source data are available online for this figure.

 © The Author(s)

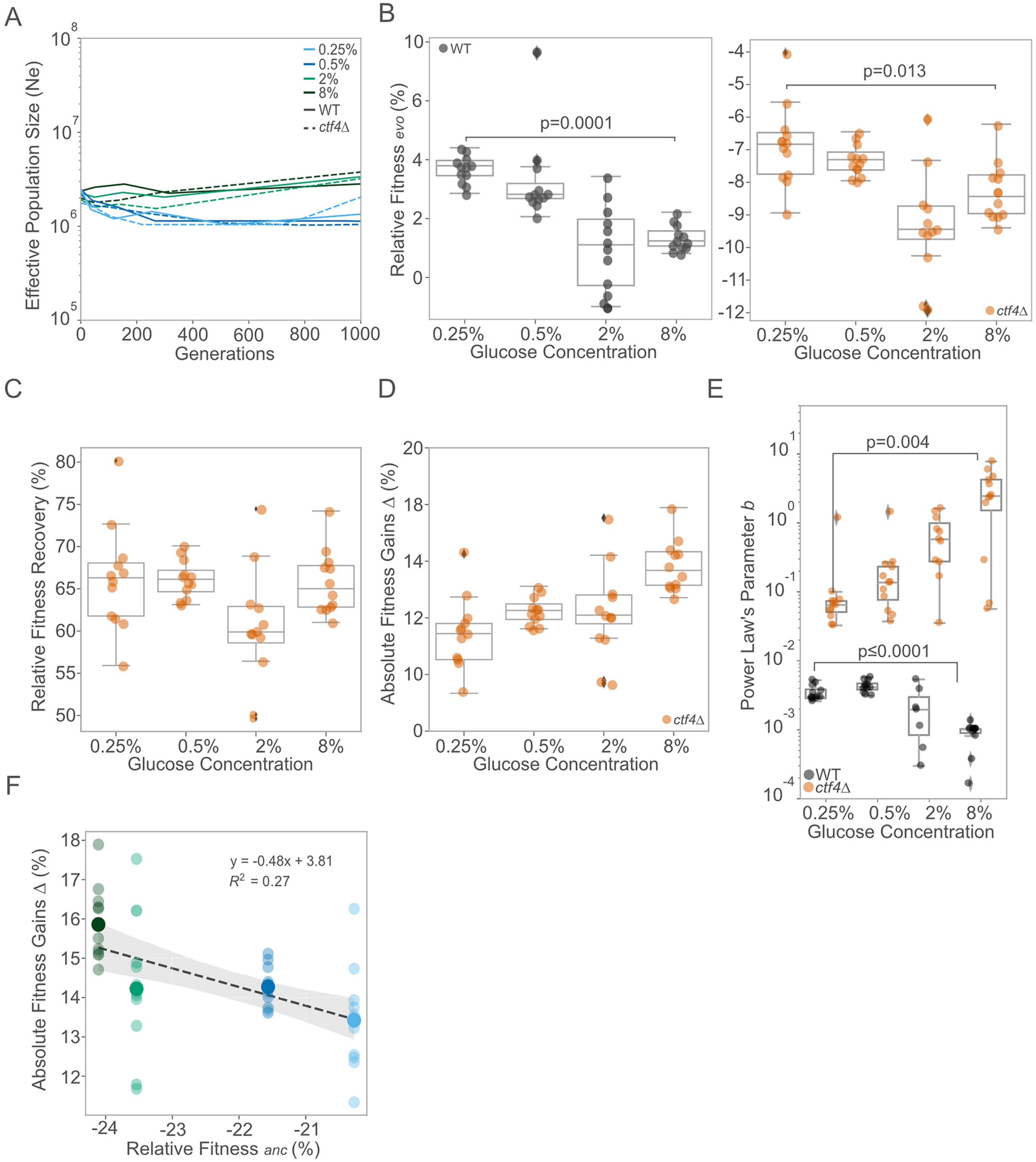
A
Effective Population Size (Ne)
Generations
0.25%
0.5%
2%
8%
WT
ctf4Δ
B
Relative Fitness evo (%)
WT
p=0.0001
p=0.013
ctf4Δ
Glucose Concentration
C
Relative Fitness Recovery (%)
Glucose Concentration
D
Absolute Fitness Gains Δ (%)
ctf4Δ
Glucose Concentration
E
Power Law's Parameter b
p=0.004
p≤0.0001
WT
ctf4Δ
Glucose Concentration
F
y = -0.48x + 3.81
R² = 0.27
Absolute Fitness Gains Δ (%)
Relative Fitness anc (%)

Figure EV2. Evolutionary dynamics under different glucose concentrations.

(**A**) Estimated Ne across generations. Solid and dashed lines represent, respectively, adjusted Ne values for WT and *ctf4Δ* populations across generations. Colors represent different glucose concentrations: light blue (0.25%), dark blue (0.5%), light green (2%), and dark green (8%). (**B**) Relative fitness at generation 1000 for evolved WT (left panel, black) and *ctf4Δ* (right panel, orange) populations ($n = 3$ replicates per population, Mann–Whitney *U* test with BH correction). (**C**) Relative fitness recovery at generation 1000 for evolved *ctf4Δ* (orange) populations. Percentage of fitness recovery was calculated by dividing the fitness gains (Δ) by the ancestral fitness ($n = 3$ replicates per population). (**D**) Absolute fitness gains (Δ) at generation 1000 for evolved *ctf4Δ* (orange) populations, per glucose concentration. Absolute fitness gains were calculated by subtracting ancestral relative fitness from evolved populations' relative fitness (Δ = evo% - anc%), both calculated as percentages relative to the same reference strain in the same glucose concentration. ($n = 3$ replicates per population). (**E**) Parameter *b* from power law fit of fitness trajectories of populations across glucose concentrations ($n = 3$ replicates per population, Mann–Whitney with BH correction, P value $= 2.19 \times 10^{-4}$). (**F**) Correlation between the absolute fitness gains (Δ) during evolution and the fitness defect of ancestor strain in each glucose concentration. Colors represent different glucose concentrations: light blue (0.25%), dark blue (0.5%), light green (2%), and dark green (8%). Pairwise comparisons were performed using the Mann–Whitney test with Bonferroni correction. Box plots in (**B–E**) represent the median (center line), 25th and 75th percentiles (lower and upper bounds of the box), and whiskers extending to the smallest and largest values within 1.5× the IQR from the lower and upper quartiles, respectively. Detailed statistical analysis and underlying data for this figure are provided in Source Data. Source data are available online for this figure.

© The Author(s)

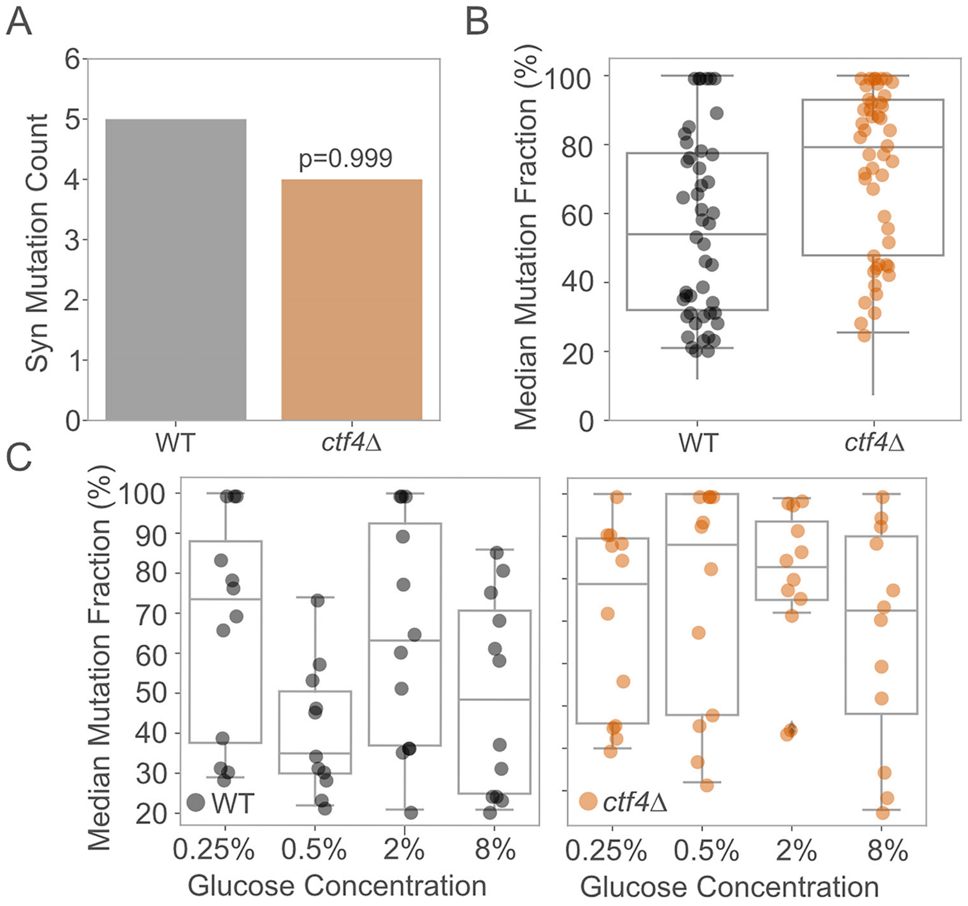

Figure EV3. Mutational counts.

(**A**) Total counts of synonymous (syn) mutations detected in evolved WT and *ctf4Δ* populations. Mann–Whitney *U* test was used to compare mutational counts. (**B**) Median mutation fraction (%) in CDS for evolved WT (black) and *ctf4Δ* (orange) populations at generation 1000 ($n = 12$ individual populations). Mann–Whitney *U* test was used to compare medians. (**C**) Median mutation fraction (%) of CDS mutations per glucose concentration, for WT (left) and *ctf4Δ* (right) at generation 1000. Statistical analysis was performed using the Mann–Whitney *U* test with BH correction. Box plots in (**B**, **C**) represent the median (center line), 25th and 75th percentiles (lower and upper bounds of the box), and whiskers extending to the smallest and largest values within 1.5× the IQR from the lower and upper quartiles, respectively. Detailed statistical analysis and underlying data for this figure are provided in Source Data. Source data are available online for this figure.

© The Author(s)

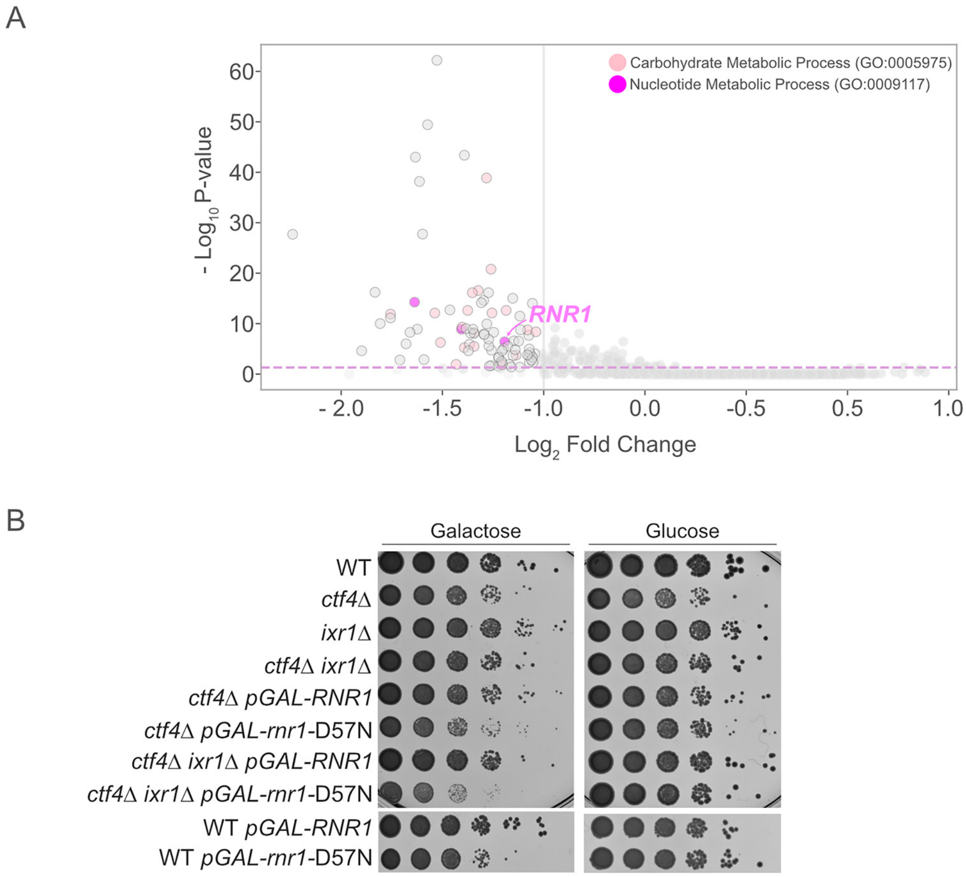

Figure EV4. Disruption of MED14 tail leads to reduced RNR1 expression.

(**A**) Volcano plot of transcriptional changes after degron-mediated removal of Med14 C-terminal (Warfield et al, 2022). Dashed purple line indicates the significance threshold (*P* value ≤ 0.01). Differentially expressed genes were identified based on two criteria: (1) an adjusted *P* value < 0.01, ensuring statistical significance, and (2) an absolute $\log_2$ fold change >1, corresponding to at least a twofold change in expression. Data analysis was performed using DESeq2. GO term enrichment analysis of downregulated genes highlights the carbohydrate (light pink) and nucleotide (magenta) metabolic processes. Detailed statistical analysis and underlying data for this figure are provided in Dataset EV1. (**B**) Overexpression of *RNR1* or its allele refractory to feedback inhibition (*rnr1*-D57N) under the Gal promoter in WT, *ctf4Δ* and *ctf4Δ ixr1Δ* backgrounds. Tenfold serial dilutions of the indicated strains were spotted onto media containing either galactose (inducing) or glucose (repressing) and incubated at 30 °C for 48 h. Overexpression of *rnr1*-D57N exacerbates the growth defects of both *ctf4Δ* and *ctf4Δ ixr1Δ*.

 © The Author(s)

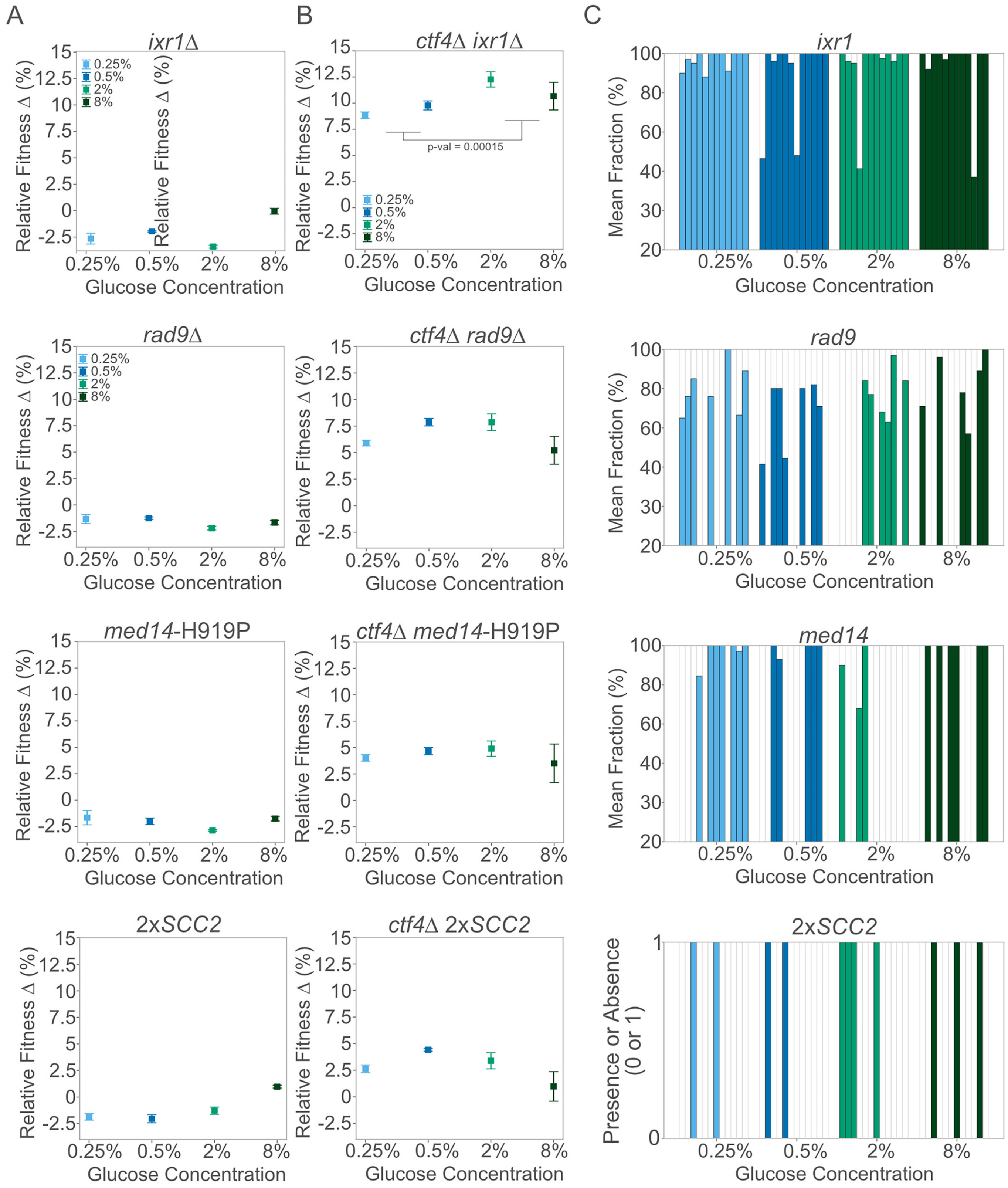
A
B
C
ixr1Δ
ctf4Δ ixr1Δ
ixr1
p-val = 0.00015
rad9Δ
ctf4Δ rad9Δ
rad9
med14-H919P
ctf4Δ med14-H919P
med14
2xSCC2
ctf4Δ 2xSCC2
2xSCC2
0.25%
0.5%
2%
8%
Relative Fitness Δ (%)
Mean Fraction (%)
Presence or Absence (0 or 1)
Glucose Concentration

Figure EV5. Fitness of reconstructed strains.

(**A**) Mean relative fitness of reconstructed putative adaptive mutations in WT background. Error bars represent SD. Colors indicate glucose concentrations: light blue (0.25%), dark blue (0.5%), light green (2%), and dark green (8%) ($n = 4$). (**B**) Changes in mean relative fitness of ancestral *ctf4*Δ clones carrying reconstructed putative adaptive mutations (Δ = |anc %|-|reconstructed %|). Error bars represent standard deviation, with errors propagated from the two fitness measurements used to calculate fitness change (Δ) ($n = 4$). (**C**) Frequencies of adaptive mutations across glucose concentrations at 1000 generations. Each bar represents the 12 parallel populations evolved in each glucose concentration, by order (1 to 12). Allele frequencies (mean fraction) in populations were derived from deep sequencing data of genomic DNA extracted from a population sample. Statistical analysis was performed using Mann–Whitney test to compare high and low glucose effect on fitness. Detailed statistical analysis and underlying data for this figure are provided in Source Data. Source data are available online for this figure.

 © The Author(s)
